# Recent advances in epilepsy genomics and genetic testing

**DOI:** 10.12688/f1000research.21366.1

**Published:** 2020-03-12

**Authors:** Malavika Hebbar, Heather C. Mefford

**Affiliations:** 1 Division of Genetic Medicine, Department of Pediatrics, University of Washington, Seattle, WA, 98105, USA

**Keywords:** Whole genome sequencing, Gene panels, Next generation sequencing, Developmental and epileptic encephalopathy, Epilepsy, Novel genes, Chromosomal microarray, Genetic testing

## Abstract

Developmental and epileptic encephalopathies (DEEs) are a group of severe, early onset epilepsies characterized by refractory seizures, developmental delay or regression associated with ongoing epileptic activity, and generally poor prognosis. DEE is genetically and phenotypically heterogeneous, and there is a plethora of genetic testing options to investigate the rapidly growing list of epilepsy genes. However, more than 50% of patients with DEE remain without a genetic diagnosis despite state-of-the-art genetic testing. In this review, we discuss the major advances in epilepsy genomics that have surfaced in recent years. The goal of this review is to reach a larger audience and build a better understanding of pathogenesis and genetic testing options in DEE.

## Background

The developmental and epileptic encephalopathies (DEEs) are a heterogeneous group of severe, early onset conditions characterized by developmental delay or regression associated with refractory seizures and generally poor prognosis ^1^. The incidence of epilepsy is nearly 70 per 100,000 children younger than 2 years and genetic epilepsies account for more than 0.4% of the general population constituting 30% of all epilepsies ^2^. The prevalence of epilepsy in the United States is 5–8 million subjects annually, while the incidence is 35–71/100,000 per year ^3^, though epidemiological data specific for DEEs are just emerging. A study on a broader group of severe epilepsies beginning before 18 months found an incidence of one in 2,000 births ^4– 6^. Some of the most well-studied DEEs include infantile spasms and Dravet, Lennox–Gastaut, and West syndromes.

Over the last decade, next-generation sequencing (NGS) has advanced the field of human genetics and genomics significantly ^7^, leading to an explosion of gene discovery across many human disorders. The number of disease-associated genes has grown to 4,132, and over 50 genes have been newly associated with epilepsy in the last three years alone ^8^. However, the new technologies have also brought new challenges ^9^. The ability to perform sequencing across large cohorts of affected individuals with variable but related phenotypes highlights “phenotype expansions” associated with some disease genes. For the epilepsies, patients can have clinical presentations that range from static to degenerative, clouding a clear distinction between isolated DEEs and secondary epilepsies associated with neurodevelopmental disorders (NDDs) ^10^. A great benefit of using NGS is its ability to deliver clinical diagnosis in a short time, but the available “cafeteria choice” of cutting-edge genetic tests can leave medical professionals and patients’ families confused.

In this review, we discuss the major advances in epilepsy genomics that have surfaced in recent years and summarize the pros and cons of genetic testing options in DEEs that could help clinicians and patients reach the end of their “diagnostic odyssey” faster and in a cost-effective way.

## Genetic testing

DEE is genetically and phenotypically heterogeneous, and there is a plethora of genetic testing options ranging from gene panels, which may include a few or hundreds of genes, to exome sequencing (ES), which investigates all ~20,000 genes. These are NGS techniques, also known as massive parallel sequencing (MPS), which include a variety of approaches that facilitate simultaneous sequencing of a large number of DNA segments ^11^. Whole ES and targeted gene panels have contributed incredibly towards novel gene discovery, particularly in the pediatric epilepsies ^12^. Sequencing all three billion bases of the genome, genome sequencing (GS), is mostly done in research settings but will inevitably enter the clinical realm soon.

Copy-number variants (CNVs) contribute significantly to variation in the human genome. CNVs are estimated to cause 1.2% difference for every reference genome ^13^. CNVs can be detected by several genomic methods including conventional karyotype (deletions/duplications >5 Mb) and chromosomal microarrays (CMA, ~100 kb–5 Mb). Other methods such as quantitative PCR and multiplex ligation-dependent probe amplification are targeted approaches to detect smaller variations (<1 kb).

The most common types of genetic causes of DEE are sequence changes, responsible for 30–40% of cases, and chromosomal deletions or duplications, responsible for 5–10% of cases ^14, 15^. Gene panels provide a higher sequencing depth and lower cost when compared to ES and GS but restrict the diagnosis to specific genes in the panel. Importantly, some large panels are based on ES, with restricted analysis of only the “panel” genes, so the benefit of higher depth of coverage is lost, but this opens up the possibility of future reanalysis to include the whole exome. ES also provides good sequencing depth at a lower cost; however, it is restricted to protein coding regions only. CNVs can be predicted by this method but require a secondary method to plot the breakpoints. Selection of the most appropriate test may depend on a variety of factors including age at seizure onset, severity of disease, other associated features, and patient insurance.

## Novel genes in DEE

Several novel genes and disorders associated with DEE have been identified in the last few years ^16– 18^ ( Table 1). Many of the genes causing epilepsy encode components of neuronal ion channels leading to neuronal hyperexcitability or depletion of inhibitory mechanisms ^19, 20^. However, recently, several new genes coding for proteins other than ion channels have been identified, such as chromatin remodelers, intracellular signaling molecules, metabolic enzymes, transcription factors, and mitochondrial complex genes ^5, 21, 22^. The search term “epilepsy” OR “seizure” OR “epileptic syndrome” OR “epileptic encephalopathy” from 2016 to 2019 led to 66 entries in Online Mendelian Inheritance in Man. Although comprehensive discussion of all the discoveries is beyond the scope of this review, selected major advances are highlighted below.

**Table 1.** Epilepsy genes and phenotypes catalogued in Online Mendelian Inheritance in Man (OMIM) since 2016.

Gene	Phenotype	OMIM phenotype #
*Chromatin remodeling*		
*ACTL6B*	Epileptic encephalopathy, early infantile, 76	#618470
*SMARCC2*	Coffin-Siris syndrome 8	#618362
*STAG2*	Neurodevelopmental disorder, X-linked, with craniofacial abnormalities	#301022
*Intracellular signaling*		
*CSF1R*	Brain abnormalities, neurodegeneration, and dysosteosclerosis	#618476
*YWHAZ*	Popov-Chang syndrome	#618428
*CHP1*	Spastic ataxia 9, autosomal recessive	#618438
*Ion channels and neurotransmitter receptors*		
*CACNA1E*	Epileptic encephalopathy, early infantile, 69	#618285
*GABRG2*	Epileptic encephalopathy, early infantile, 74	#618396
*CACNA2D2*	Cerebellar atrophy with seizures and variable developmental delay	#618501
*HCN1*	Generalized epilepsy with febrile seizures plus, type 10	#618482
*CACNA1B*	Neurodevelopmental disorder with seizures and nonepileptic hyperkinetic movements	#618497
*KCNK4*	Facial dysmorphism, hypertrichosis, epilepsy, intellectual/developmental delay, and gingival overgrowth syndrome	#618381
*SLC25A42*	Metabolic crises, recurrent, with variable encephalomyopathic features and neurologic regression	#618416
*ATP1A1*	Hypomagnesemia, seizures, and mental retardation 2	#618314
*SLC28A1*	Uridine-cytidineuria	#618477
*SCN8A*	Myoclonus, familial, 2	#618364
*SLC9A7*	Intellectual developmental disorder, X-linked 108	#301024
*Metabolism*		
*GLS*	Epileptic encephalopathy, early infantile, 71	#618328
*PARS2*	Epileptic encephalopathy, early infantile, 75	#618437
*RNF13*	Epileptic encephalopathy, early infantile, 73	#618379
*FCSK*	Congenital disorder of glycosylation with defective fucosylation 2	#618324
*PPP3CA*	Arthrogryposis, cleft palate, craniosynostosis, and impaired intellectual development	#618265
*PPP2CA*	Neurodevelopmental disorder and language delay with or without structural brain abnormalities	#618354
*MTHFS*	Neurodevelopmental disorder with microcephaly, epilepsy, and hypomyelination	#618367
*P4HTM*	Hypotonia, hyperventilation, impaired intellectual development, dysautonomia, epilepsy, and eye abnormalities	#618493
*DHPS*	Neurodevelopmental disorder with seizures and speech and walking impairment	#618480
*MAST1*	Mega-corpus-callosum syndrome with cerebellar hypoplasia and cortical malformations	#618273
*DEGS1*	Leukodystrophy, hypomyelinating, 18	#618404
*MYORG*	Basal ganglia calcification, idiopathic, 7, autosomal recessive	#618317
*ALKBH8*	Intellectual developmental disorder, autosomal recessive 71	#618504
*NAXD*	Encephalopathy, progressive, early onset, with brain edema and/or leukoencephalopathy, 2	#618321
*KDM6B*	Neurodevelopmental disorder with coarse facies and mild distal skeletal abnormalities	#618505
*HS6ST2*	Paganini-Miozzo syndrome	#301025
*TRMT1*	Intellectual developmental disorder, autosomal recessive 68	#618302
*COLGALT1*	Brain small vessel disease 3	#618360
*IREB2*	Neurodegeneration, early-onset, with choreoathetoid movements and microcytic anemia	#618451
*PIGB*	Epileptic encephalopathy, early infantile, 80	#618580
*Mitochondrial metabolism*		
*MICOS13*	Combined oxidative phosphorylation deficiency 37	#618329
*GFM2*	Combined oxidative phosphorylation deficiency 39	#618397
*Neuronal development*		
*NFASC*	Neurodevelopmental disorder with central and peripheral motor dysfunction	#618356
*NHLRC2*	Fibrosis, neurodegeneration, and cerebral angiomatosis	#618278
*Nucleoplasmic transport*		
*NUP133*	Galloway-Mowat syndrome 8	#618349
*NUP214*	Susceptibility to acute infection-induced encephalopathy 9	#618426
*Regulation of cell morphology and motility*		
*BICD2*	Spinal muscular atrophy, lower extremity-predominant, 2b, prenatal onset, autosomal dominant	#618291
*DOCK3*	Neurodevelopmental disorder with impaired intellectual development, hypotonia, and ataxia	#618292
*PHACTR1*	Epileptic encephalopathy, early infantile, 70	#618298
*MACF1*	Lissencephaly 9 with complex brainstem malformation	#618325
*DYNC1I2*	Neurodevelopmental disorder with microcephaly and structural brain anomalies	#618492
*Synaptic vesicle cycle*		
*NEUROD2*	Epileptic encephalopathy, early infantile, 72	#618374
*MAPK8IP3*	Neurodevelopmental disorder with or without variable brain abnormalities	#618443
*Transcriptional regulation*		
*ATN1*	Congenital hypotonia, epilepsy, developmental delay, and digital anomalies	#618494
*RORB*	Susceptibility to idiopathic generalized epilepsy 15	#618357
*ZNF142*	Neurodevelopmental disorder with impaired speech and hyperkinetic movements	#618425
*RSRC1*	Intellectual developmental disorder, autosomal recessive 70	#618402
*TCF20*	Developmental delay with variable intellectual impairment and behavioral abnormalities	#618430
*EIF3F*	Intellectual developmental disorder, autosomal recessive 67	#618295
*ZBTB11*	Intellectual developmental disorder, autosomal recessive 69	#618383
*CNOT1*	Holoprosencephaly 12 with or without pancreatic agenesis	#618500
*NFIB*	Macrocephaly, acquired, with impaired intellectual development	#618286
*SOX4*	Coffin-Siris syndrome 10	#618506
*TRRAP*	Developmental delay with or without dysmorphic facies and autism	#618454
*Others Transmembrane protein*		
*TMEM94*	Intellectual developmental disorder with cardiac defects and dysmorphic facies	#618316
*Structural protein*		
*COL3A1*	Polymicrogyria with or without vascular-type Ehlers–Danlos syndrome	#618343
*Nuclear DNA polymerase*		
*POLE*	Intrauterine growth retardation, metaphyseal dysplasia, adrenal hypoplasia congenita, genital anomalies, and immunodeficiency	#618336
*Multiple functions*		
*WDR4*	Microcephaly, growth deficiency, seizures, and brain malformations	#618346
*Intracellular trafficking*		
*TRAPPC2L*	Encephalopathy, progressive, early onset, with episodic rhabdomyolysis	#618331

ES trios have revealed the influence *of de novo* mutations as a genetic cause of severe epilepsies ( Table 1). A recent study compared *de novo* variants identified in individuals with variable NDDs with and without epilepsy ^23^. In the subset of 1,942 subjects with NDDs with epilepsy, 33 genes were observed to have significant excess of *de novo* variants, three of which had limited or no previous evidence of disease association: *CACNA1E*, *SNAP25*, and *GABRB2*. Nine *de novo* missense and two truncating variants in *CACNA1E* variants were identified in this cohort ^23^. In a subsequent study, *de novo* variants in *CACNA1E* were identified in 30 individuals with DEE ^16^. Detailed phenotyping revealed refractory infantile-onset seizures, severe hypotonia, and profound developmental delay, often with congenital contractures, hyperkinetic movement disorders, macrocephaly, and early death ^16^. Functional analysis revealed consistent gain-of-function effects in R-type calcium channels. Some patients were seizure free on treatment with the anti-epileptic drug topiramate, which blocks R-type calcium channels. The condition is now catalogued as early infantile epileptic encephalopathy type 69 (#MIM 618285).

The *RORB* gene, which encodes the retinoid-related nuclear receptor ROR-beta, was recently associated with photosensitive generalized epilepsy in a large family segregating a nonsense variant in the gene ^24^. In the same study, two individuals with *de novo* coding variants in *RORB* and a third individual with a *de novo* intragenic deletion presented with significant developmental delays and behavioral abnormalities in addition to their generalized epilepsy, consistent with a diagnosis of DEE. Together, these results suggest that *RORB* haploinsufficiency causes a fairly consistent epilepsy phenotype but variable developmental outcomes.

Several additional recent discoveries highlight the overlap between DEEs and NDDs, with several new genes associated with syndromic epilepsy, including *NBEA*, *FBXO11*, and *SMARCC2* ^25– 27^. *NBEA* has long been a candidate gene for NDDs and autism ^28^. Clear disease association and description of the phenotypic spectrum were recently reported after the identification of 24 *de novo* variants in patients with NDD, many of whom also had generalized epilepsy. Similarly, one-quarter to one-third of individuals with pathogenic variants in *FBXO11* or *SMARCC2*, each associated with variable NDD, also have epilepsy.

Recessive genes are a rare but important cause of DEE. Inborn errors of metabolism and malformations of cortical development constitute most of the autosomal recessive epilepsies ^29^. Glycosylphosphatidylinositol (GPI) anchored proteins play key roles in the human body, mainly in development and neurogenesis. Several genes involved in GPI biosynthesis and the remodeling pathway are causative of autosomal recessive epilepsy. One such gene that was recently identified is *PIGB* ^30^. This group reported 16 patients from 10 unrelated families with early infantile epileptic encephalopathy, type 80 (#MIM 618580). Some other recessive epileptic encephalopathies are due to *WWOX*, *TBC1D24*, *UBA5*, and *SLC13A5* ^31– 34^. *TBC1D24* is known to cause a continuum of features that were originally described as distinct, recognized Mendelian phenotypes ranging from autosomal dominant deafness to autosomal recessive epileptic encephalopathy ^35^. Similarly, in addition to causing epileptic encephalopathy type 28 (#MIM 616211), *WWOX* is implicated as the molecular basis of spinocerebellar ataxia, type 12 (#MIM 614322) ^36, 37^. Both these genes are examples of a spectrum of disorders with increasingly blurred lines differentiating them as more individuals and pathogenic variants are identified. Recently, homozygous pathogenic variants in *CSF1R*, encoding a tyrosine kinase growth factor receptor for colony-stimulating factor-1, were identified in patients with brain abnormalities, neurodegeneration, and dysosteosclerosis ^38^. This gene was previously implicated in a dominant adult-onset leukoencephalopathy. Proliferation and growth of macrophages, including microglia, require colony-stimulating factor-1 receptor (CSF1R). This study represents an under-recognized group of genes that are associated with well-described, dominant phenotypes but can also produce a different clinical picture when present in biallelic, recessive states. This is important for filtering and interpreting variants from NGS data, as candidate variants cannot be eliminated based on poor phenotypic fit ^39^.

## CNVs in DEE

Studies using CMA have shown that pathogenic CNVs account for 5–10% of childhood epilepsies including DEE ^40– 42^, and CMA is the recommended first-line genetic test if the clinical picture includes dysmorphism, intellectual disability, congenital anomalies, and other neuropsychiatric features ^43^. However, NGS is increasingly being employed in the detection of CNVs. One good example is the detection of deletions in *TANGO2*. TANGO proteins play a crucial role in redistributing Golgi membranes into the endoplasmic reticulum ^44^. Bi-allelic *TANGO2* pathogenic variants have been identified as a cause of a pediatric condition with multi-organ involvement ^45^. Recently, a study identified intragenic, multi-exon deletions in *TANGO2* by reanalysis of ES data ^45, 46^. The most common disease-causing allele (55%) in one series was deletion of exons 3–9 of *TANGO2* ^17^. ES is not yet a match for CMA for CNV detection, as it can provide data about only the protein coding or exonic regions, but it is an increasingly powerful diagnostic tool, and a growing number of algorithms are being developed to aid the detection of CNVs by NGS. With the introduction of ES and GS, it is now possible to detect both single nucleotide variations and CNVs using an exome- or genome-wide approach with a single test ^47^.

## Future of epilepsy genomics

Despite state-of-the-art genetic testing, more than 50% of patients with DEE remain without a genetic diagnosis. Whole GS is increasingly being used to uncover the role of non-coding genetic material in the human genome ^48, 49^. Undoubtedly, massively parallel sequencing has greatly accelerated disease gene (and variant) discovery, but most studies and nearly all clinical testing employ gene panels or ES, limiting the genomic search space and the types of variants that can potentially be identified. For disorders like fragile X syndrome that are due to the expansion of triplet repeats, testing strategies other than gene panels or exome are required. Several studies have proposed a genetic testing strategy to achieve the highest clinical utility, cost-effectiveness, and diagnostic yield for individuals with epilepsy ^50– 52^, but specific testing algorithms are likely to change over time as new tests are introduced and the costs of existing tests decrease. New assays may be required to detect lesser-known but important molecular mechanisms.

Post-zygotic, somatic mosaic mutations are increasingly identified as an important cause of genetic disorders ^22, 53^. In epilepsies, many of the mutations in the mTOR pathway that lead to brain malformations are somatic mosaic mutations. Typically, leukocyte-derived DNA is used in individuals with DEE to search for germline variants, which are inherited or arise *de novo* in the zygote. Recent studies have demonstrated that post-zygotic somatic variants also underlie DEE ^22, 54– 58^ but can be easily missed by standard NGS tests.

Another field that has potential to uncover some of the underlying molecular mechanisms is epigenetics. Epimutations represent a class of mutational event where the epigenetic status of a genomic locus deviates significantly from the normal state ^59^. Methylation of DNA and histone modifications are increasingly being implicated as causative or contributing factors for several conditions ^60, 61^. DNA methylation at CpG dinucleotides is the most widely studied epigenetic modification. Methylation represents an epigenetic change—a chemical modification of DNA that does not change the underlying DNA sequence. A recent study investigated the role of *de novo* methylation changes in NDDs using methylation chips ^62^. In a cohort of 489 affected individuals, of which 16% had epilepsy, the authors identified rare differential methylation in 23% of cases when compared to controls. When the parents were able to be tested, ~40% of the methylation variants were *de novo*, suggesting that *de novo* methylation abnormalities may be causative in 5–10% of their cohort. When identified, the underlying causes of the methylation changes were varied and included CNVs, sequence variants in regulatory elements, or repeat expansions, each of which is easily missed by conventional (even next-generation) sequencing methods. In a second study of undiagnosed NDDs using a similar approach ^63^, candidate differentially methylated regions in two individuals with epilepsy and intellectual disability of unknown etiology were identified.

Several techniques that enable longer read lengths (up to 200 kb), such as nanopore-based “fourth-generation” sequencing ^64^ and single molecule, real time (SMRT) sequencing ^65^, have recently emerged. The advantages of long reads include shorter sequencing time, ability to sequence AT- or GC-rich regions and repeat stretches, and the detection of large structural abnormalities including insertions, deletions, inversions, translocations, and tandem/interspersed regions ^66, 67^.

## Conclusion

NGS-based technologies are a mainstay of clinical diagnostic testing, and the applications and testing options will only increase as the technology, bioinformatics, and resources evolve. NGS successfully detects single nucleotide variations, structural rearrangements, and CNVs. Clinical phenotypes are now being defined by the underlying molecular basis. Interpretation of NGS data is an iterative process involving forward genetics along with a reverse phenotyping approach. The dynamic nature of data analysis should be explained to patients and their families. As more and more novel genetic and epigenetic etiologies are unveiled in DEE, the challenge for clinical and research laboratories is to make sure the testing is clinically relevant, is cost effective, and can be integrated into clinical care.
